# Otud6b induces pulmonary arterial hypertension by mediating the Calpain-1/HIF-1α signaling pathway

**DOI:** 10.1007/s00018-024-05291-3

**Published:** 2024-06-15

**Authors:** Yu Liu, Bailin Tang, Hongxin Wang, Meili Lu

**Affiliations:** 1 Key Laboratory of Cardiovascular and Cerebrovascular Drug Research of Liaoning Province, Jinzhou Medical University, Jinzhou, China; 2 School of Pharmacy, Harbin Medical University, Harbin, China; 3 Tongji Medical College of Basic Sciences, Huazhong University of Science and Technology, Wuhan, China

**Keywords:** Pulmonary hypertension, Otud6b, Calpain-1, HIF-1α, Proteomic

## Abstract

Pulmonary hypertension (PAH) is a cardiopulmonary disease in which pulmonary artery pressure continues to rise, leading to right heart failure and death. Otud6b is a member of the ubiquitin family and is involved in cell proliferation, apoptosis and inflammation. The aim of this study was to understand the role and mechanism of Otud6b in PAH. C57BL/6 and Calpain-1 knockout (KO) mice were exposed to a PAH model induced by 10% oxygen. Human pulmonary artery endothelial cells (HPACEs) and human pulmonary artery smooth muscle cells (HPASMCs) were exposed to 3% oxygen to establish an in vitro model. Proteomics was used to determine the role of Otud6b and its relationship to Calpain-1/HIF-1α signaling. The increased expression of Otud6b is associated with the progression of PAH. ROtud6b activates Otud6b, induces HIF-1α activation, increases the production of ET-1 and VEGF, and further aggravates endothelial injury. Reducing Otud6b expression by tracheal infusion of siOtud6b has the opposite effect, improving hemodynamic and cardiac response to PAH, reducing the release of Calpain-1 and HIF-1α, and eliminating the pro-inflammatory and apoptotic effects of Otud6b. At the same time, we also found that blocking Calpain-1 reduced the effect of Otud6b on HIF-1α, and inhibiting HIF-1α reduced the expression of Calpain-1 and Otud6b. Our study shows that increased Otud6b expression during hypoxia promotes the development of PAH models through a positive feedback loop between HIF-1α and Calpain-1. Therefore, we use Otud6b as a biomarker of PAH severity, and regulating Otud6b expression may be an effective target for the treatment of PAH.

**Graphical Abstract:**

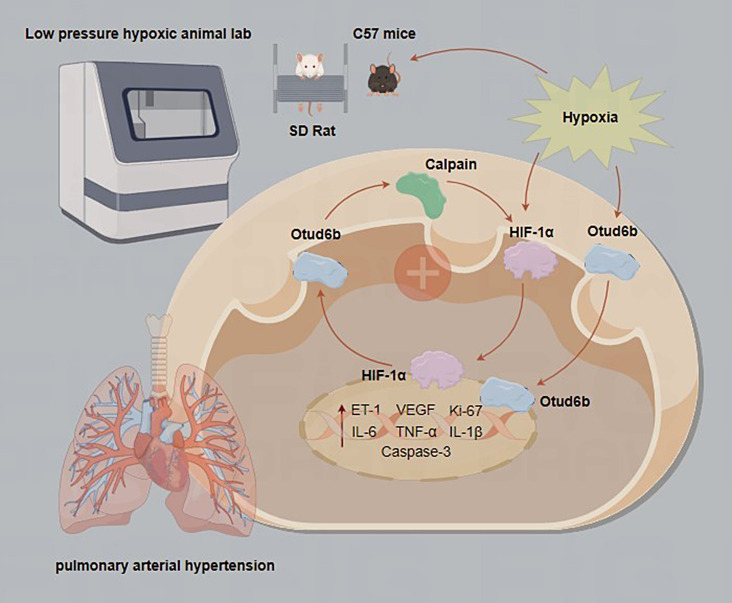

**Supplementary Information:**

The online version contains supplementary material available at 10.1007/s00018-024-05291-3.

## Introduction

Pulmonary arterial hypertension (PAH) is characterized by pulmonary vascular remodeling, which causes progressive occlusive vascular lesions in the distal pulmonary artery circulation, leading to right ventricular failure and death [1, 2]. The inducing factors of PAH are diverse, and hypoxia is one of the important risk factors for pulmonary artery remodeling [3]. The literature indicates that the initiation and/or progression of PAH and the process of pulmonary vascular remodeling are accompanied by changes in perivascular inflammation, fibrosis, and the proliferation and apoptosis characteristics of cell [4–6]. However, due to the complex pathogenesis of PAH and unclear mechanism research, the therapeutic effect of PAH is not ideal, and the prognosis is not significantly improved.

OTU is one of the most important members of DUB, which has been shown to regulate cellular cascade signaling and is strongly closely associated with inflammation and cancer [7–9]. OTU deubiquitinase 6B (Otud6b) affects cell proliferation by binding protein-activated complexes and plays a key role in many biological processes such as cell cycle regulation, apoptosis, inflammation, and DNA repair [10–15]. Wang et al. [11] showed that Otud6b reduces angiogenesis of atherosclerotic plaques, enhances plaque stability and delays the progression of atherosclerosis by regulating the proliferation, migration and lumen formation of endothelial cells. MiKi et al. [10] showed that Otud6b-AS1 silencing significantly reduced the proliferation and apoptosis of HPASMCs. According to the results of previous proteomic studies in our laboratory, the expression of Otud6b protein in the lung tissue of hypoxia induced PAH mice was significantly increased in the hypoxia group compared with the normal group. Compared with hypoxia group, hypoxia + Calpain-1 knockout group not only improved the pathological changes of PAH, but also significantly reduced the protein expression of Otud6b, suggesting that Calpain-1 plays an important role in the regulation of Otud6b.

Calpain is a conservative family of calcium dependent cysteine proteases that are commonly expressed in all cells [16, 17]. At least 16 types of Calpain have been described, of which Calpain-1 and Calpain-2 have the most prominent characteristics, consisting of a large subunit with catalytic activity of 80 kD and a small subunit with regulatory activity of 28 kD [18, 19]. According to previous research results in our laboratory [20, 21], Calpain-1 mediates vascular remodeling and fibrosis through HIF-1α in hypoxia pulmonary arterial hypertension. Calpain-1 plays an important role in PAH by regulating HIF-1α. However, whether Calpain-1 is involved in Otud6b mediated PAH has not been reported in the literature.

Hypoxia-inducible factor-1α (HIF-1α) is a transcription factor that is activated under hypoxia conditions and consists of β and α subunits [22]. Under hypoxia conditions, HIF-1α subunits are stable due to inhibition of Prolyl Hydroxylase Domain Proteins (PHDs) and therefore accumulate in the nucleus. HIF-1α binds to hypoxia responsive elements and regulates the transcription of hundreds of genes involved in different processes, such as erythropoiesis, angiogenesis, metabolic reprogramming, cell proliferation, and apoptosis/survival, in response to hypoxia [23–25]. However, there are no relevant literature reports on whether there is a correlation between Calpain-1, HIF-1α and Otud6b. Therefore, this article aims to investigate whether there is a link between HIF-1α and Calpain-1/Otud6b signaling pathway in hypoxia induced PAH.

## Results

### The expression of Otud6b in lung tissues of C57BL/6 mice was increased by hypoxia

**Fig. 1:**
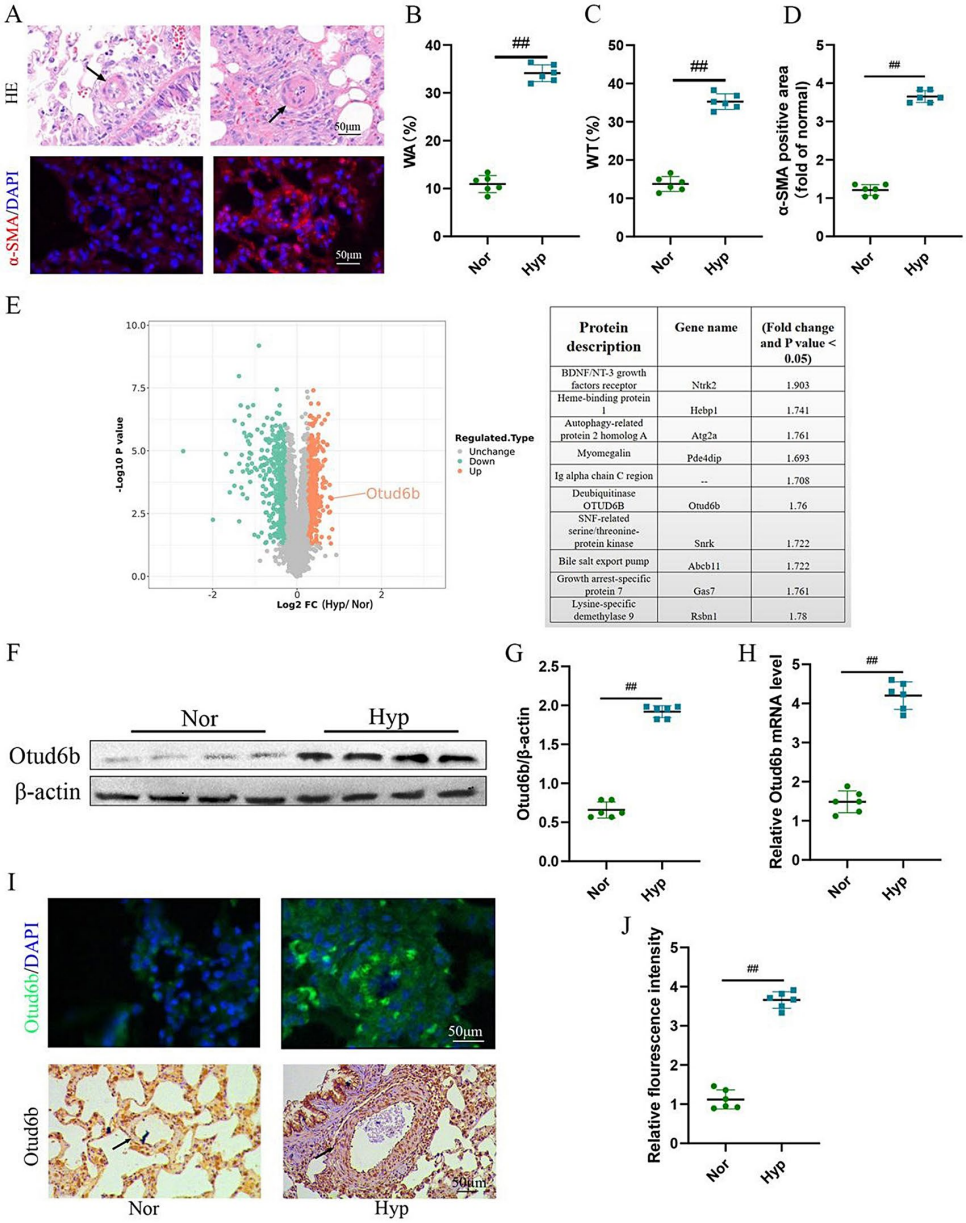
The expression of Otud6b in lung tissues of C57BL/6 mice was increased by hypoxia. Representative images of H&E staining (**A-C**) and α-SMA immunostaining (**A-D**) of pulmonary arterioles in Nor and Hypoxia mice. The ratio of vascular wall area to total vascular area, the ratio of vascular wall thickness to total vascular thickness, and the positive area of α-SMA immunostaining were used as indicators to quantify the pulmonary arteriole thickness. **(E)** The volcano map of differentially expressed genes between hypoxia lung tissue and normal lung tissue showed that the expression of Otud6b increased under hypoxia condition compared with normal group. **(F-G)** Representative western blots of Otud6b protein levels in lung tissue from hypoxia and normal. **(H)** Total RNA was extracted from mice lung tissue, and the Otud6b mRNA level was analyzed by RT-qPCR. **(I-J)** Representative micrograph of Otud6b expression in normal and hypoxia lung tissue. Otud6b (green) and the nucleus (blue) are simultaneously stained. *n* = 6. The data are expressed as the means ± SEM. ^##^*P <* 0.01 vs. the Nor group

We detected Otud6b expression levels in a hypoxia induced PAH mice model. As shown in the Fig. 1A-D, H&E staining and immunofluorescence staining showed that the distal medial wall of the pulmonary artery in C57BL/6 mice was significantly thickened, and the expression of α-SMA positive region was significantly increased under hypoxia conditions. These results indicate that the PAH model has been established. To further investigate protein expression in PAH, we analyzed a secondary mass spectrometry database (Mus_musculus_10090_SP_20201214.fasta) of 28-day lung tissue samples from mice induced by hypoxia. Mass spectrometry analysis showed that the protein expression was in the form of Log2. With *P* ≤ 0.05 as the standard, the change of differential expression greater than 1.3 was the significantly up-regulated change threshold. As shown in Fig. 1E, compared with Normal group, there were 511 proteins with higher expression levels (orange region) and 482 proteins with lower expression levels (green region) in PAH model. We listed the top 10 highly expressed proteins (as shown in the table) and the results showed an increase in Otud6b expression. Next, we used western blot (Fig. 1F-G) and RT-qPCR (Fig. 1H) to analyze the expression level of Otud6b in hypoxia induced C57BL/6 mice lung tissues. Compared with Nor group, hypoxia treatment significantly increased the expression levels of Otud6b protein and mRNA, which was consistent with the proteomic results. This was also confirmed by immunohistochemical staining and immunofluorescence staining of the distal pulmonary artery (Fig. 1I-J). Therefore, our results indicate increased Otud6b expression in hypoxia induced PAH mice models.

### Otud6b expression decreased in Calpain-1 KO mice lung tissues induced by hypoxia

**Fig. 2:**
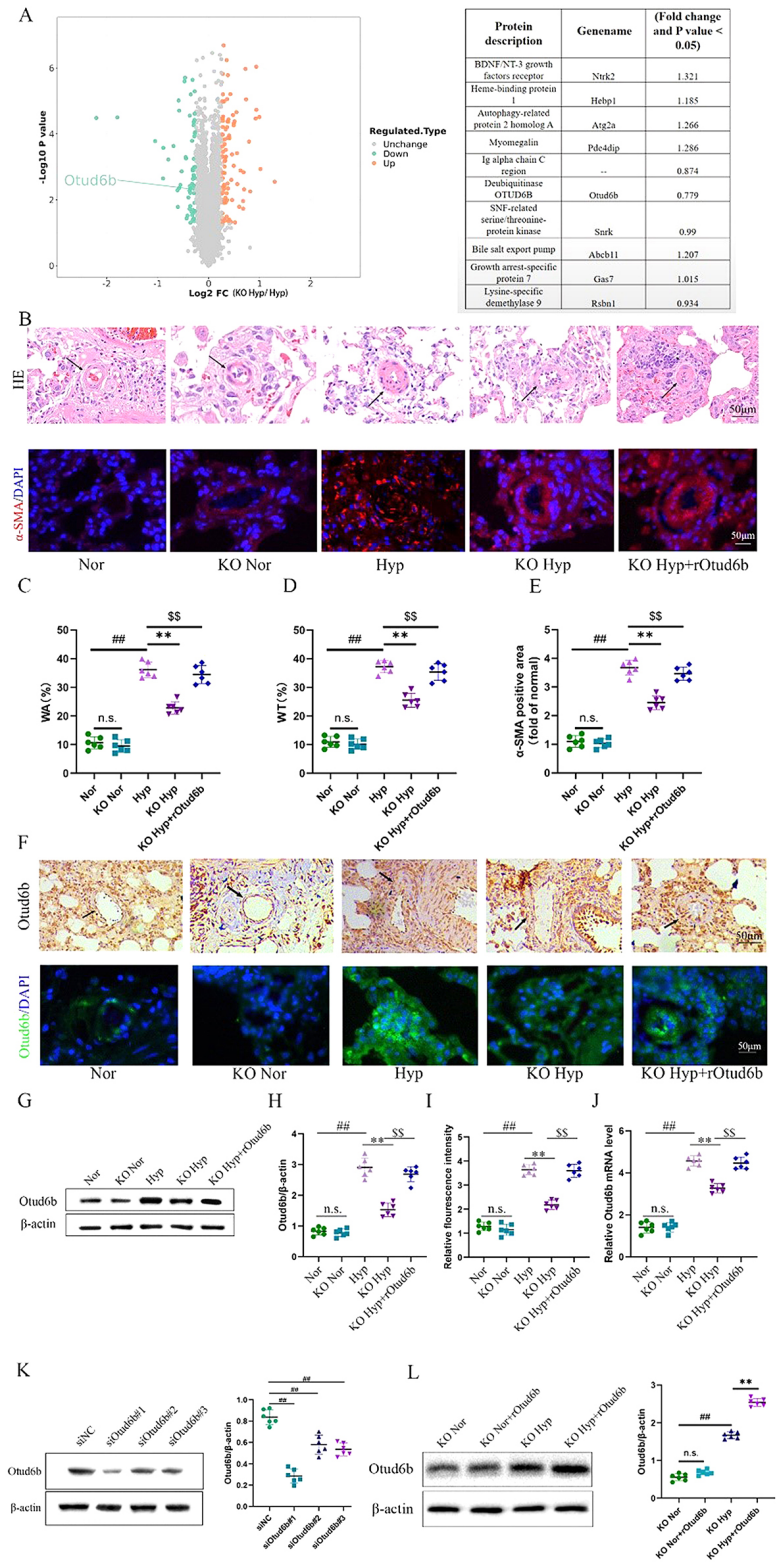
Otud6b expression decreased in Calpain-1 KO mice lung tissues induced by hypoxia. **(A)** The differentially expressed gene volcano map of hypoxia lung tissue and hypoxia knockout lung tissue showed that Otud6b expression decreased in the model knockout group compared with the model group. Representative images of H&E staining (**B-D**) and α-SMA immunostaining (**B-E**) of pulmonary arterioles in Nor, KO Nor, Hypoxia, KO Hyp, KO Hyp + rOtud6b mice. The ratio of vascular wall area to total vascular area, the ratio of vascular wall thickness to total vascular thickness, and the positive area of α-SMA immunostaining were used as indicators to quantify the pulmonary arteriole thickness. **(F, I)** Representative micrograph of Otud6b expression in Nor, KO Nor, Hypoxia, KO Hyp, KO Hyp + rOtud6b lung tissue. Otud6b(green) and the nucleus (blue) are simultaneously stained. Representative western blots of Otud6b protein levels in lung tissue **(G-H, L)** and HPAECs **(K)** of different groups. **(J)** Total RNA was extracted from mice lung tissue, and the Otud6b mRNA level was analyzed by RT-qPCR. *n* = 6. The data are expressed as the means ± SEM. Not significant (n.s.), ^##^*P <* 0.01 vs. the Nor/siNC/KO Nor group, ^**^P < 0.01 vs. the Hypoxia/KO Hyp group, ^$$^*P <* 0.01 vs. the KO Hyp group

As shown in the Fig. 2A, proteomic analysis results showed that compared with Hypoxia group, the expression of Otud6b in the lung tissue of Calpain-1 KO mice was decreased. H&E staining and immunofluorescence staining showed that compared with Hypoxia group, Calpain-1 KO mice did not show any morphological changes such as vascular wall thickening, lumen narrowing, and α-SMA positive expression area increase (Fig. 2B-E). Meanwhile, western blot (Fig. 2G-H), RT-qPCR (Fig. 2J), immunohistochemical staining and immunofluorescence staining were used to analyze the distal pulmonary artery of mice in each group (Fig. 2F, I). The results further showed that compared with Hypoxia group, the expression of Otud6b in Calpain-1 KO state was decreased, and the hypoxia induced PAH was improved.

In order to further verify the role of Otud6b in the in vivo model, recombinant protein Otud6b was intraperitoneally injected into Calpain-1 KO mice, and western blot results showed (Fig. 2L) that compared with the KO Hypoxia group, the expression of Otud6b protein was significantly increased in KO Hyp + rOtud6b group, and suggest that rOtud6b was effective in the lung. Meanwhile, compared with the KO Hypoxia group, a series of morphological changes such as vascular wall thickening, lumen narrowing and α-SMA positive expression rate were observed in the KO Hyp + rOtud6b group (Fig. 2B-E). Our experimental results showed that rOtud6b reversed the effect of Calpain-1 KO on Otud6b and aggravated the ameliorative effect of Calpain-1 KO on PAH in hypoxia mice models by increasing the expression of Otud6b protein.

### Otud6b deficiency inhibits PAH development in hypoxia mice models

As shown in the Fig. 2K, Western blot detected the knock-down effect of siOtud6b in HPAECs, siOtud6b#1 (GGAATGAAGAACGCCGTT), siOtud6b#2 (GGATCAGCTAAGAGAACAA) and siOtud6b#3 (GCTCTGTCTCACATCTTAA) had statistically significant knock-down effects compared with the siNC group. The knock-down effect of siOtud6b#1 is stronger than siOtud6b#2 and siOtud6b#3.

**Fig. 3:**
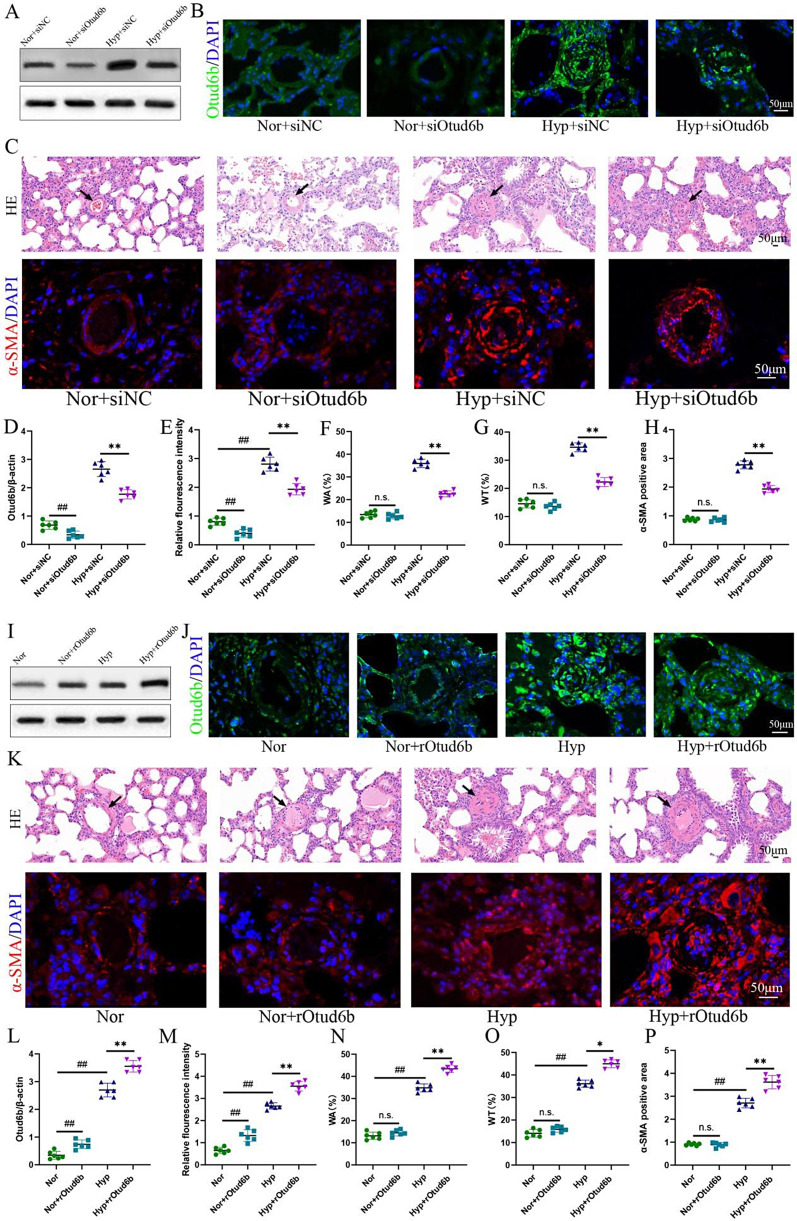
Otud6b deficiency inhibits PAH development in hypoxia mice models. **(A, D, I, L)** Representative western blots of Otud6b protein levels in lung tissue of different groups. **(B, E, J, M)** Representative micrographs of Otud6b expression in different groups of lung tissue. Otud6b (green) and the nucleus (blue) are simultaneously stained. **(C, F-H, K, N-P)** Representative images of H&E staining and α-SMA immunostaining of pulmonary arterioles in different groups of mice. The ratio of vascular wall area to total vascular area, the ratio of vascular wall thickness to total vascular thickness, and the positive area of α-SMA immunostaining were used as indicators to quantify the pulmonary arteriole thickness. *n* = 6. The data are expressed as the means ± SEM. Not significant (n.s.), ^##^*P <* 0.01 vs. the Nor / Nor + siNC group, ^*^P < 0.05, ^**^P < 0.01 vs. the Hypoxia/ Hyp + siNC group

To test whether the reduction of Otud6b inhibits PAH development, we targeted Otud6b (siOtud6b, GGGAATGAAGAACGCCGTT) and non-targeted control (siNC) modified anti-siRNA oligonucleotides by trachea infusion for 3 weeks. Western blot, and immunofluorescence staining showed that the expression of Otud6b protein in lung tissues of mice treated with siOtud6b decreased significantly (Fig. 3A-B, D-E). Both H&E and immunofluorescence staining showed that siOtud6b treatment reversed hypoxia induced vascular remodeling, including vascular wall thickening, lumen narrowing, and α-SMA positive expression area compared with siNC treatment (Fig. 3C, F-H). To further verify these results, we performed intraperitoneal injection of rOtud6b to detect the above indicators. In contrast to the siOtud6b, the expression level of Otud6b protein was significantly increased in the rOtud6b treated group (Fig. 3I-J, L-M). The above mentioned indexes were significantly increased, which induced morphological changes and further aggravated PAH (Fig. 3K, N-P). These results suggest that Otud6b may be a key target for mitigating the progression of pulmonary vascular remodeling in PAH.

**Fig. 4:**
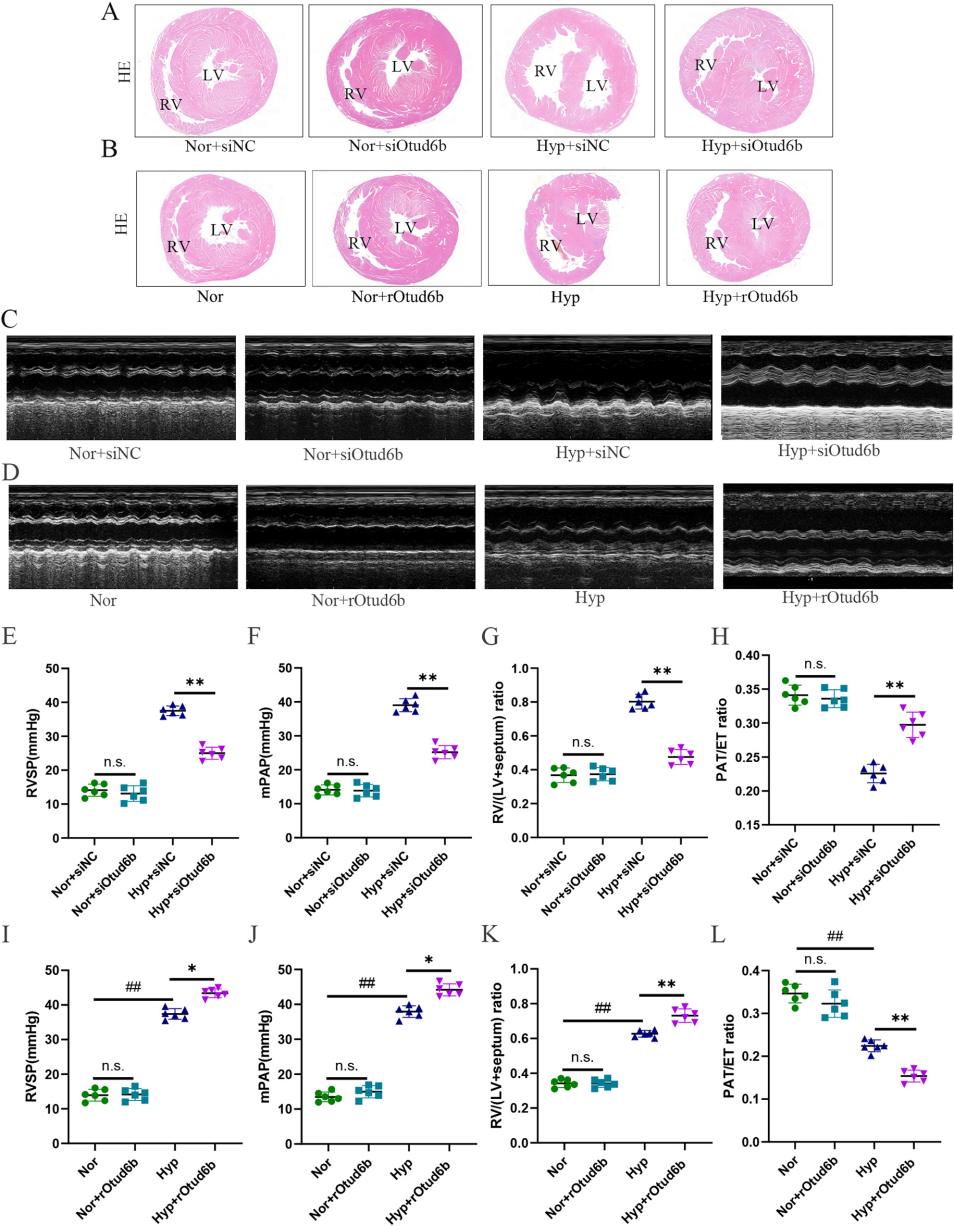
Otud6b deficiency inhibits PAH development in hypoxia mice models. **(A-B, G, K)** The representative images of cardiac histomorphology in each group of mice with H&E staining. **(C-D, H, L)** Echocardiographic analysis showed that the PAT/ET ratio of hypoxia-induced PAH mice model was decreased, and the PAT/ET ratio of rOtud6b mice was significantly decreased. In mice injected with siOtud6b in the trachea, the situation was reversed. **(E-F, I-J)** RVSP, mPAP images showed that Otud6b deficiency inhibits PAH development in hypoxia mice models. *n* = 6. The data are expressed as the means ± SEM. Not significant (n.s.), ^##^*P <* 0.01 vs. the Nor group, ^*^P < 0.05, ^**^P < 0.01 vs. the Hypoxia/ Hyp + siNC group

In addition, under normal conditions, there were no significant differences in right ventricular systolic blood pressure (RVSP), mean pulmonary artery pressure (mPAP), and the ratio of right ventricular (RV) weight to left ventricular (LV) plus diaphragm weight (RV/LV + S) between Nor + siNC and Nor + siOtud6b groups. However, PAH and hypertrophy were successfully induced in the Hyp + siNC group, but pulmonary artery pressure and ventricular hypertrophy were significantly reversed in the Hyp + siOtud6b group (Fig. 4A, E-G). The results of echocardiography showed that the PAT/ET ratio of the Hyp + siNC group decreased significantly, while the decline of the above mentioned indexes was reversed in the Hyp + siOtud6b group (Fig. 4C, H). To further verify the role of Otud6b, we tested the above indexes by intraperitoneal injection of rOtud6b. The results showed that only Hypoxia group and Hyp + rOtud6b group showed significant increase in hemodynamic indexes (Fig. 4I-J), ventricular hypertrophy (Fig. 4B, K) and PAT/ET ratio decreased (Fig. 4D, L), while Nor group and Nor + rOtud6b group did not. Our data suggest that rOtud6b can aggravate PAH development, while inhibiting Otud6b expression can reverse PAH development.

### Inhibition of Otud6b has therapeutic effect on PAH

**Fig. 5:**
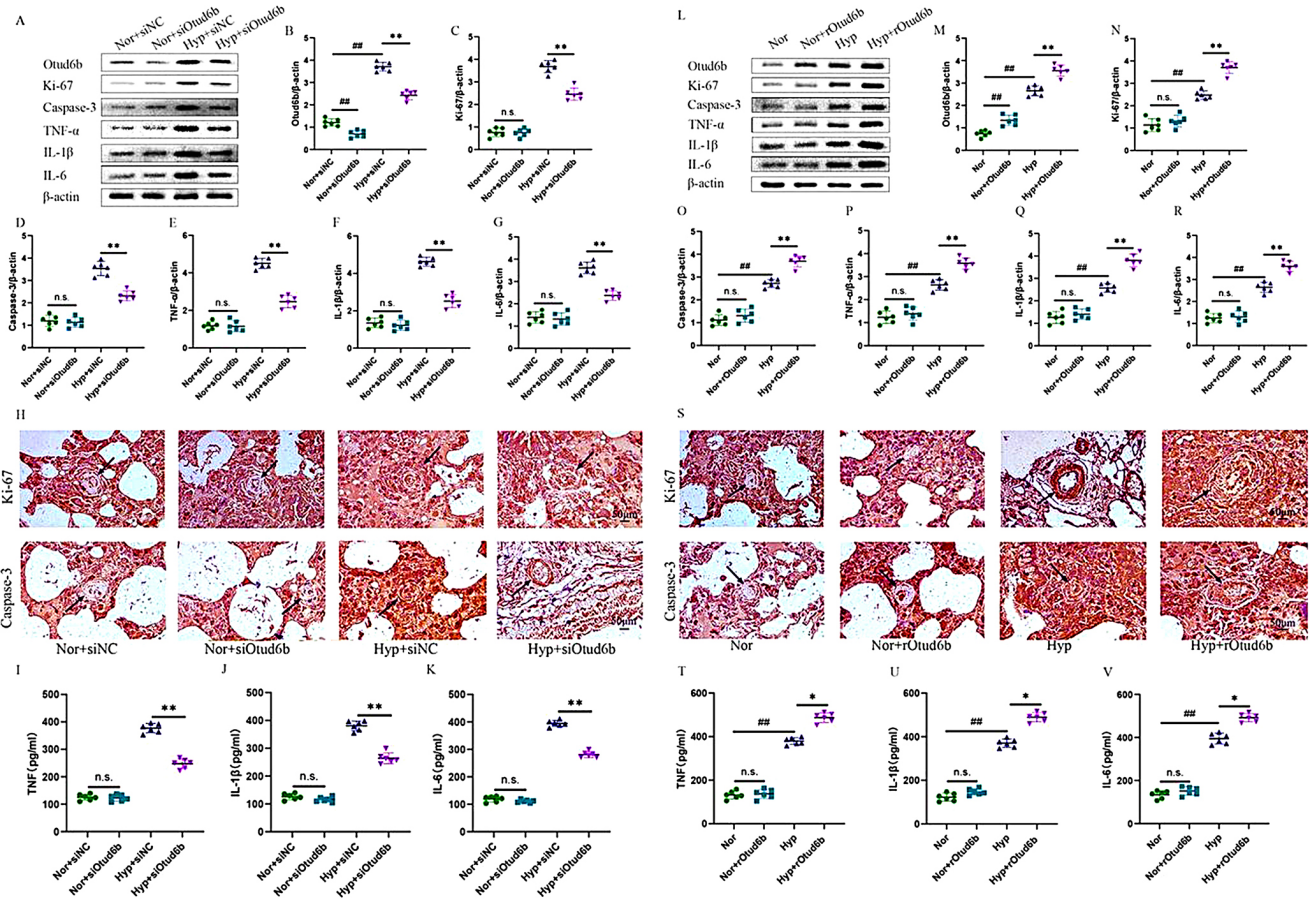
Inhibition of Otud6b has therapeutic effect on PAH. **(A-G, L-R)** Representative western blots of Otud6b, Ki-67, Caspase-3, TNF-α, IL-1β and IL-6 proteins levels in lung tissue of different groups. **(H, S)** The expressions of Ki-67 and Caspase-3 in pulmonary arterioles were detected by immunohistochemical staining. **(I-K, T-V)** ELISA assay showed that siOtud6b reduced the release of TNF-α, IL-1β and IL-6 in the lung tissue of hypoxia induced PAH mice, while rOtud6b reversed this result. *n* = 6. The data are expressed as the means ± SEM. Not significant (n.s.), ^##^*P <* 0.01 vs. the Nor group, ^*^P < 0.05, ^**^P < 0.01 vs. the Hypoxia /Hyp + siNCgroup

To detect the effect of Otud6b on PAH, we examined inflammation, apoptosis, and proliferative phenotypes in mice treated with siOtud6b and rOtud6b. As shown Fig. 5A-G, compared with the siNC group, the expression levels of apoptotic protein Caspase-3, inflammatory factors TNF-α, IL-1β and IL-6 proteins in the siOtud6b treatment group were significantly reduced, and the expression levels of proliferative protein Ki-67 were significantly increased (Fig. 5H-K). In contrast to the siOtud6b study, compared with the Hypoxia group, the expression levels of apoptotic protein Caspase-3, inflammatory factors TNF-α, IL-1β and IL-6 were significantly increased in the Hyp + rOtud6b group, while the expression levels of proliferative protein Ki-67 were significantly decreased (Fig. 5L-V). Our data clearly showed that rOtud6b further aggravated hypoxia induced cellular inflammation, apoptosis and proliferation, while siOtud6b reversed the progression of rOtud6b, aggravated PAH and had a protective effect on PAH.

### Increased Otud6b expression in HPAECs and HPASMCs of hypoxia induced vitro model

**Fig. 6:**
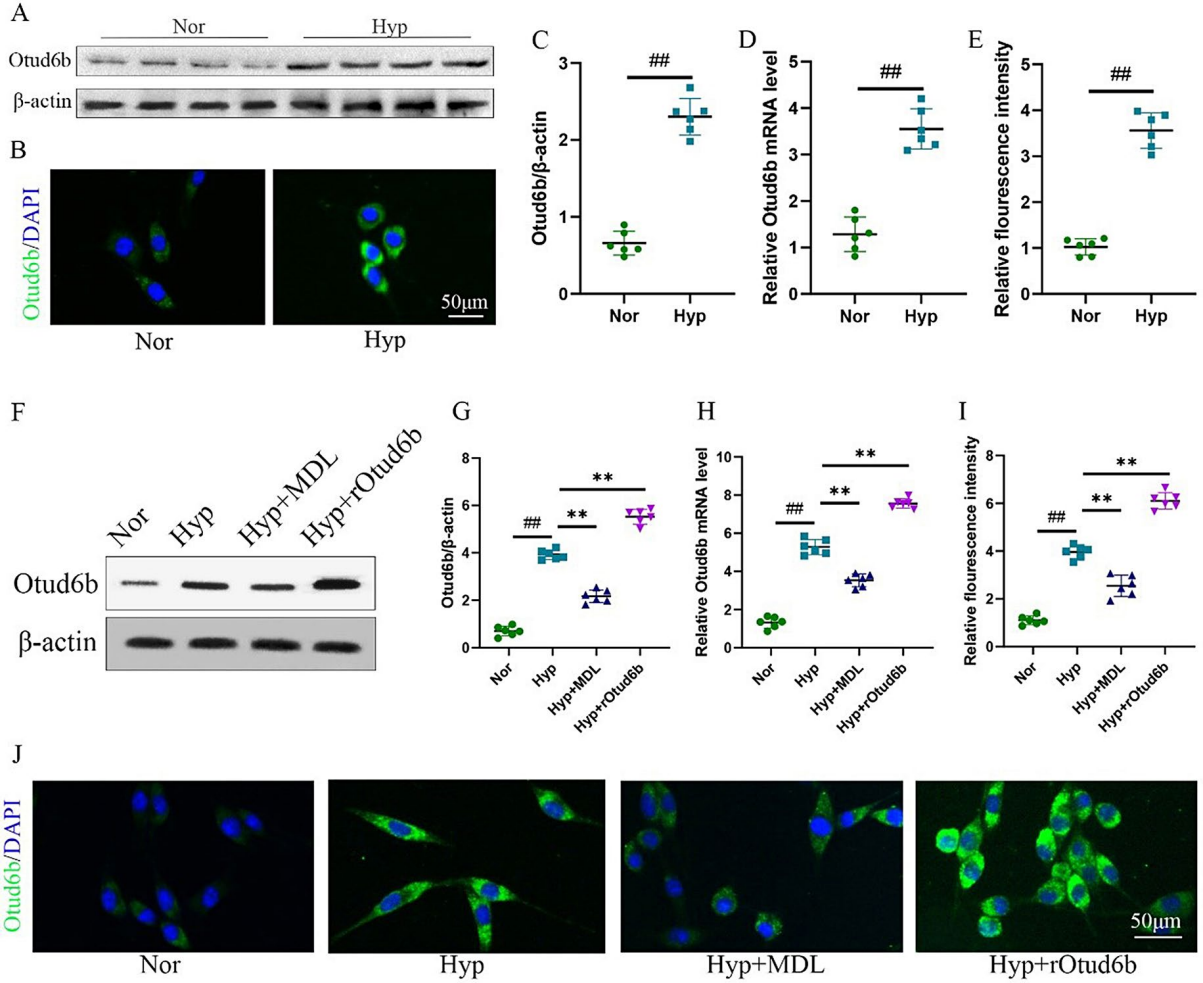
Calpain-1 inhibitor reduce the expression of Otud6b in HPAECs and HPASMCs of hypoxia induced vitro model. **(A, C, F, G)** Representative western blots of Otud6b protein levels in different HPAECs groups. **(B, E, I-J)** Representative images of Otud6b immunostaining of different groups in HPAECs. Otud6b (green) and the nucleus (blue) are simultaneously stained. **(D, H)** HPAECs were stimulated at 3% oxygen concentration for 24 h. Total RNA was extracted and the mRNA level of Otud6b was analyzed by RT-qPCR. *n* = 6. The data are expressed as the means ± SEM. ^##^*P <* 0.01 vs. the Nor group, ^**^P < 0.01 vs. the Hypoxia group

**Fig. 7:**
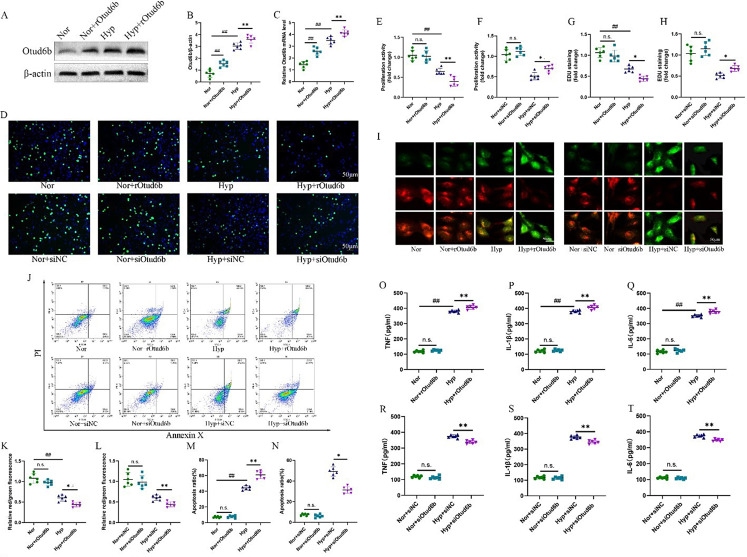
Phenotypic effects of Otud6b in HPAECs and HPASMCs. **(A-B)** Western blot was used to detect the expression of Otud6b protein in HPAECs by hypoxia. **(C)** Total RNA was isolated and the mRNA level of Otud6b was analyzed by RT-qPCR. **(D, G-H)** Representative EDU staining images showed the proliferative activity of HPAECs treated with rOtud6b or PBS for 24 h under either normoxia or hypoxia conditions. **(E-F)** CCK-8 assay detection of cell viability. **(I, K-L)** HPAECs cells were stained with JC-1, and the mitochondrial membrane potential was evaluated by fluorescence microscopy. **(J, M-N)** Flow cytometry showed the effect of siOtud6b and rOtud6b on apoptosis of HPAECs under normal oxygen and hypoxia conditions. **(O-T)** The expression of TNF-α, IL-1β and IL-6 in HPAECs induced by siOtud6b and rOtud6b under normal or hypoxia conditions was detected by ELISA assay. *n* = 6. The data are expressed as the means ± SEM. Not significant (n.s.), ^##^*P <* 0.01 vs. the Nor group, ^**^P < 0.01 vs. the Hypoxia/ Hyp + siNC group

To investigate the expression level of Otud6b in vitro, we treated HPAECs and HPASMCs with hypoxia (3% oxygen) to establish in vitro models. The results of RT-qPCR, western blot and immunofluorescence staining showed that the mRNA and protein expression levels of Otud6b in HPAECs and HPASMCs were significantly increased under hypoxia conditions, and the increase of Otud6b was mainly located in the cytoplasm of cells (Figs. 6A-E and 7A-C).

### Calpain-1 inhibitor reduce the expression of Otud6b in HPAECs and HPASMCs of hypoxia induced vitro model

In vitro, we introduced the Calpain-1 inhibitor MDL-28,170 to study the expression of Otud6b under hypoxia condition. The results of RT-qPCR (Figs. 6H and 7F), western blot (Fig. 6F-G) and immunofluorescence staining (Figs. 6I-J and 7D-E) showed that the Calpain-1 inhibitor MDL could reduce the mRNA and protein expression levels of Otud6b in HPAECs and HPASMCs compared with the Hypoxia group. To further verify the role of Otud6b, we treated hypoxia induced HPAECs and HPASMCs with the rOtud6b. Compared with Hypoxia group, the mRNA and protein expression levels of Otud6b in Hyp + rOtud6b group were significantly increased.

### Phenotypic effects of Otud6b in HPAECs and HPASMCs

**Fig. 8:**
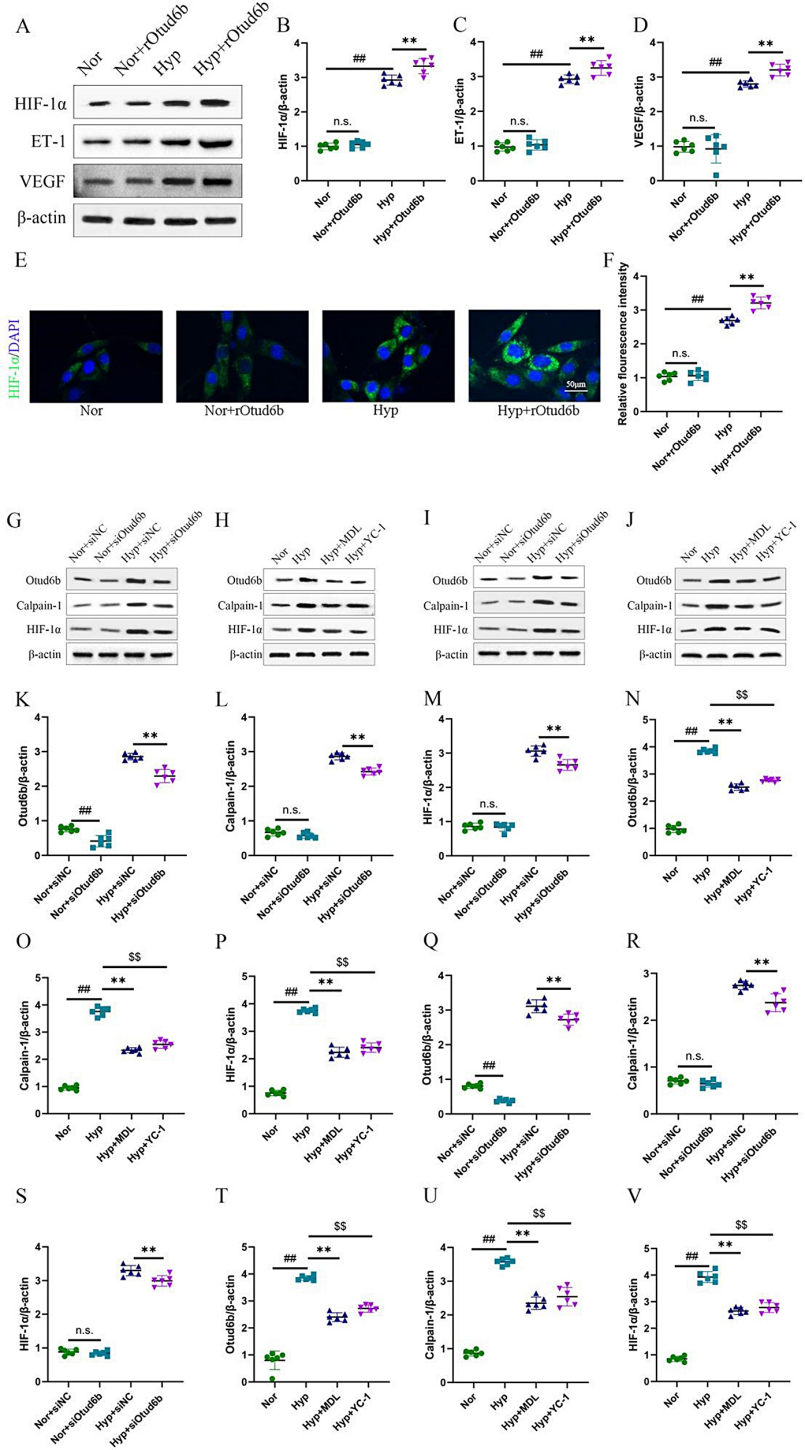
A positive feedback loop exists between Otud6b and HIF-1α signal pathways in hypoxia. **(A-D, G-V)** Representative western blots of Otud6b, HIF-1α, Calpain-1, ET-1 and VEGF proteins levels in different HPAECs groups. **(E-F)** Representative images of HIF-1α immunostaining of different groups in HPAECs. HIF-1α (green) and the nucleus (blue) are simultaneously stained. *n* = 6. The data are expressed as the means ± SEM. Not significant (n.s.), ^##^*P <* 0.01 vs. the Nor/ Nor + siNC group, ^**^P < 0.01 vs. the Hypoxia/ Hyp + siNC group, ^$$^*P <* 0.01 vs. the Hypoxia group

**Fig. 9:**
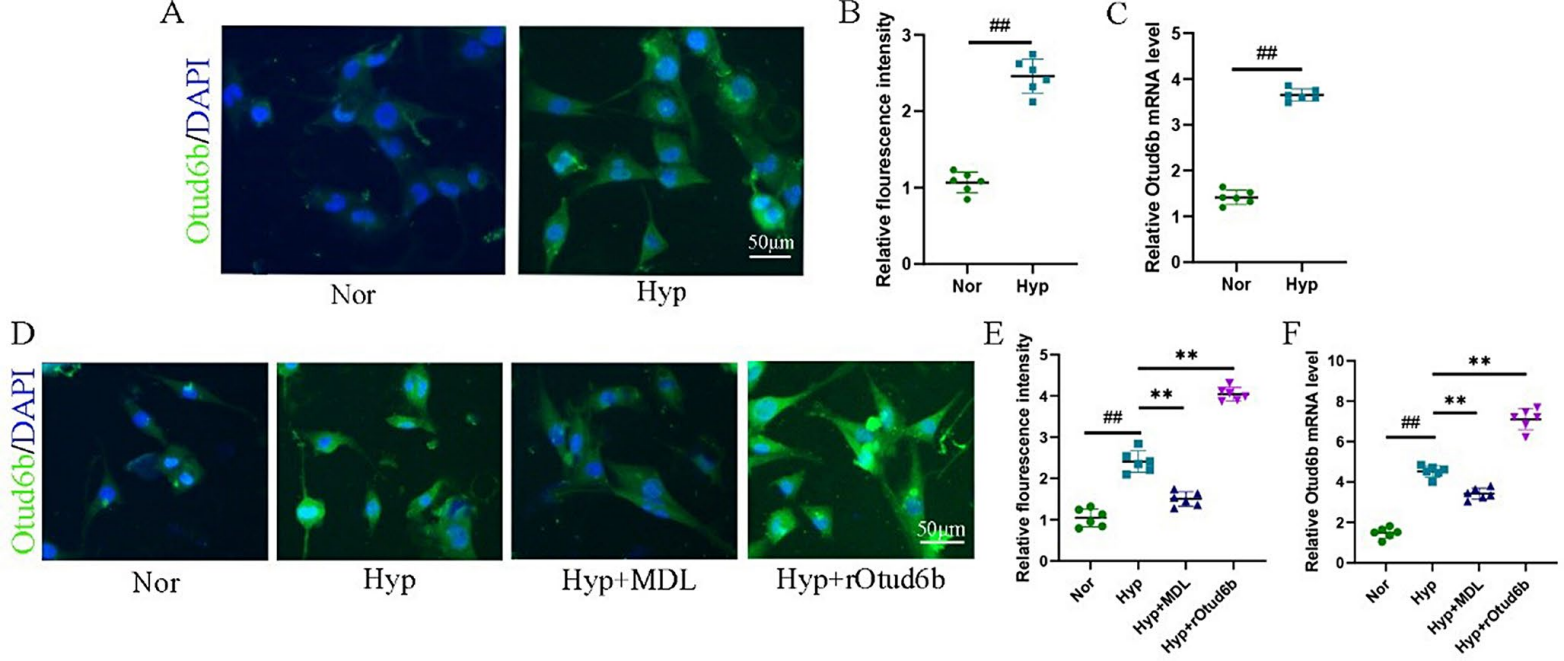
Calpain-1 inhibitor reduce the expression of Otud6b in HPAECs and HPASMCs of hypoxia induced vitro model. **(A-B, D-E)** Representative images of Otud6b immunostaining of different groups in HPASMCs. Otud6b (green) and the nucleus (blue) are simultaneously stained. **(C, F)** HPASMCs were stimulated at 3% oxygen concentration for 24 h. Total RNA was extracted and the mRNA level of Otud6b was analyzed by RT-qPCR. *n* = 6. The data are expressed as the means ± SEM. ^##^*P <* 0.01 vs. the Nor group, ^**^P < 0.01 vs. the Hypoxia group

We treated HPAECs and HPASMCs with rOtud6b and exposed it to hypoxia (3% O_2_) for 24 h to observe the effect of Otud6b on HPAECs and HPASMCs. Western blot and RT-qPCR results showed that the Otud6b protein level in HPAECs of rOtud6b treatment group was significantly increased (Fig. 8A-C). The results of CCK-8 experiment showed that rOtud6b significantly reduced the proliferation rate of HPAECs compared with PBS treated cells (Fig. 8E). EDU staining also showed that after rOtud6b treatment, the proliferation rate of HPAECs was significantly reduced, while that of HPASMCs was significantly increased (Figs. 8D and G and 9A-B). To further confirm these results, we conducted proliferation experiments after siOtud6b knockout in HPAECs and HPASMCs. The experimental results showed that, contrary to the results of rOtud6b, CCK-8 assay and EDU staining showed that the proliferation rate of HPAECs was significantly increased after transfection with siotud6b, while the proliferation rate of HPASMCs was significantly decreased (Figs. 8D, F and H and 9A-C).

The results of JC-1 mitochondrial membrane potential staining showed that the mitochondrial membrane potential of HPAECs treated by rOtud6b was significantly decreased compared with that of PBS treated cells (Fig. 8I, K). Flow cytometry showed that the apoptosis rate of HPAECs treated with rOtud6b was significantly increased (Fig. 8J, M). Immunofluorescence staining showed that the expression of pro-apoptotic protein Caspase-3 was significantly decreased in rOtud6b treated HPASMCs (Fig. 9D-E). At the same time, apoptosis was also determined in HPAECs and HPASMCs treated with siOtud6b. Contrary to the results of rOtud6b study, the apoptosis rate of cells transfected with siOtud6b was significantly reduced, and the pro-apoptotic protein Caspase-3 was significantly increased (Figs. 8I, J, L and N and 9D-F).

ELISA results showed that the expression levels of TNF-α, IL-6 and IL-1β in rOtud6b-treated HPAECs and HPASMCs were significantly higher than those in PBS-treated cells (Figs. 7O-Q and 9G-I). Contrary to the results of the rOtud6b study, the expression of inflammatory factors in the cells transfected with siOtud6b was significantly reduced (Figs. 7R-T and 9J-L). These results suggest that Otud6b may aggravate HPAECs and HPASMCs inflammation. Therefore, our findings provide further evidence that short-term in vitro induction of Otud6b protein is sufficient to cause HPAECs and HPASMCs to exhibit many important functional abnormalities associated with PAH pathogenesis.

### Otud6b increases HPAECs production of HIF-1α, ET-1 and VEGF

Liu et al. showed that Otud6b can interact with HIF-1α to coordinate various cellular process responses under hypoxia. To explore the role of Otud6b in HIF-1α-mediated function during hypoxia, we examined the protein expression of HIF-1α, ET-1 and VEGF in HPAECs. Western blot (Fig. 8A-D) and immunofluorescence staining (Fig. 9E-F) showed that HIF-1α protein expression increased in HPAECs treated with hypoxia and rOtud6b. Our data suggest that Otud6b may exacerbate the HPAECs response to acute or chronic hypoxia. Consistent with changes in HIF, Otud6b overexpression significantly increased the production of ET-1 and VEGF in HPAECs.

### A positive feedback loop exists between Otud6b and HIF-1α signal pathways in hypoxia

**Fig. 10:**
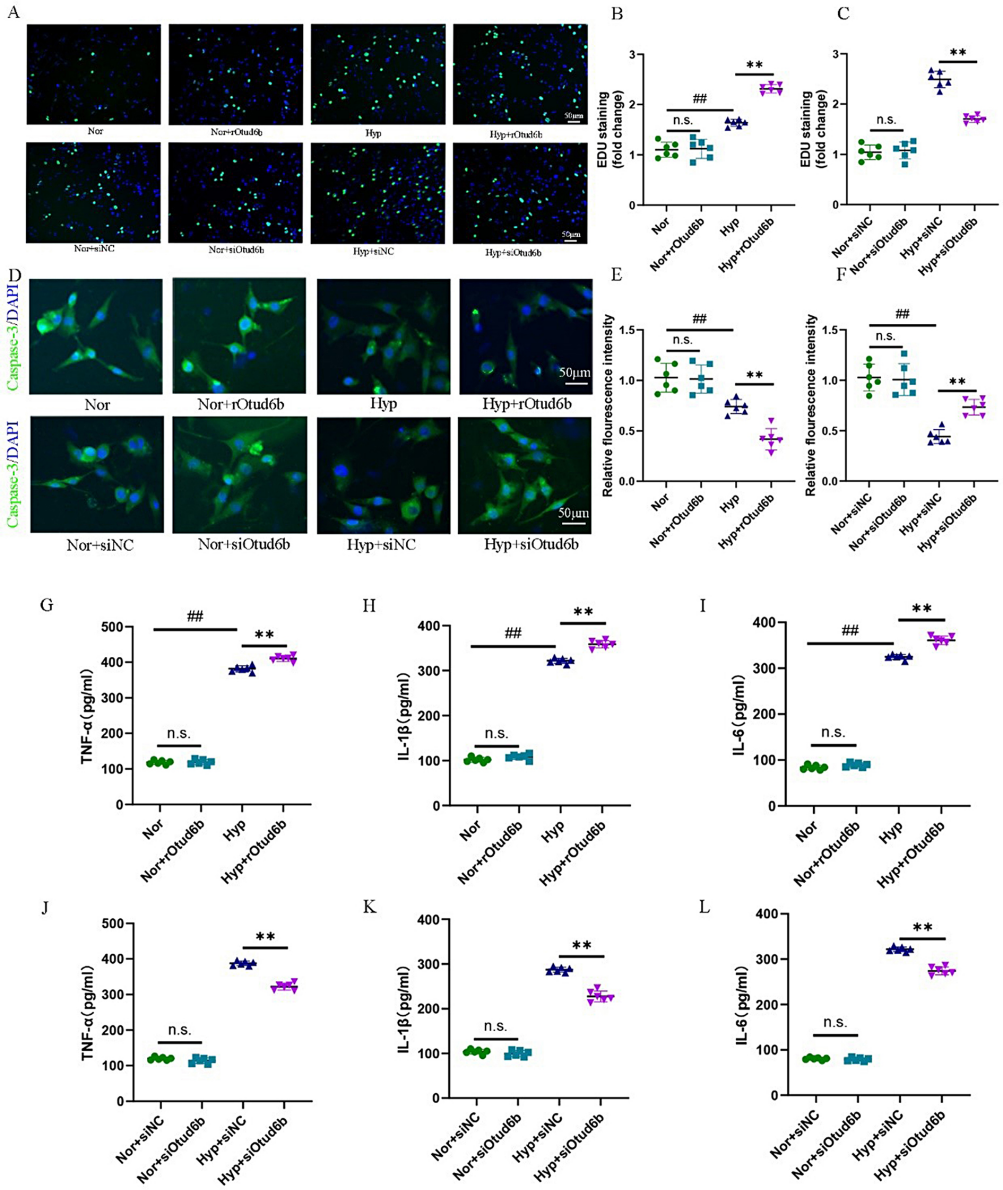
Phenotypic effects of Otud6b in HPAECs and HPASMCs. **(A-C)** Representative EDU staining images showed the proliferative activity of HPASMCs treated with rOtud6b or PBS for 24 h under either normal or hypoxia conditions. **(D-F)** The expression of Caspase-3 was detected by immunofluorescence staining. Caspase-3 (green) and the nucleus (blue) are simultaneously stained. **(G-L)** The expression of TNF-α, IL-1β and IL-6 in HPASMC induced by siOtud6b and rOtud6b under normal or hypoxia conditions was detected by ELISA assay. *n* = 6. The data are expressed as the means ± SEM. Not significant (n.s.), ^##^*P <* 0.01 vs. the Nor group, ^**^P < 0.01 vs. the Hypoxia/ Hyp + siNC group

Previous studies in our laboratory have shown that Calpain-1 and HIF-1α inhibit each other to improve hypoxia induced pulmonary vascular remodeling and fibrosis. Our results also suggest that Otud6b can mediate HIF-1α protein expression. To determine the interaction among Calpain-1, Otud6b, and HIF-1α, we examined hypoxia induced mice and HPAECs in vivo and in vitro experiments. As shown in the figure, expressions of Calpain-1, Otud6b, and HIF-1α proteins were increased in hypoxia induced mice and HPAECs, while not seen in siOtud6b treated mice and HPAECs. In addition, blocking the protein level of Calpain-1 with the Calpain-1 inhibitor MDL also weakened the effect of Otud6b on HIF-1α. In addition, inhibition of HIF-1α with HIF-1α inhibitor YC-1 reduced protein levels of Calpain-1 and Otud6b (Fig. 10G-V). These results suggest a positive feedback loop between Otud6b and Calpain-1/HIF-1α signaling.

## Discussion

Pulmonary arterial hypertension (PAH) is a rare disease characterized by poor remodeling of the arterial tree, resulting in increased vascular resistance followed by increased right ventricular afterload, and eventually progression to heart failure [26–29]. There are many causes of pulmonary hypertension, and hypoxia is the main inducement factor of PAH [30–34]. PAH can cause RVSP and mPAP abnormalities, increased RV/(LV + S), and histomorphologic changes such as WA% and WT%. At the same time, HPAECs and HPASMCs can also cause phenotypic changes such as inflammation, proliferation and apoptosis. We developed both in vivo and in vitro models to simulate the pathological features of human PAH. The results showed that in both models, the expression of Otud6b in pulmonary vascular was upregulated, and in addition, Otud6b gene knock-down attenuated PAH development. Our results suggest that inhibiting Otud6b is an important approach to the treatment of PAH. At the same time, we observed a difference between the proteomic results and the immunofluorescence results. We analyzed that the results were different due to the whole organ is used, the differences observed by proteomics are somewhat small. For immunofluorescence and western blot experiments, samples are taken from different mice, so there was a substantial quantitative difference between the results obtained using mass spectrometry and immunoblots and immunofluorescence.

Vascular endothelial lesions are thought to occur early in the pathogenesis of PAH in animal models and humans [35]. We found that activation of Otud6b expression with rOtud6b increased endothelial cell inflammation and apoptosis, smooth muscle cells inflammation and proliferation. An increase in Otud6b in the cytoplasm was observed in hypoxia HPAECs and HPASMCs, suggesting that HPAECs and HPASMCs derived Otud6b may play a role in regulating the cell’s response to hypoxia. Under the induction of hypoxia, Otud6b accumulates in the cytoplasm of endothelial cells and smooth muscle cells, leading to endothelial cell dysfunction and smooth muscle cell proliferation, further inducing and aggravating the inflammation and apoptosis of endothelial cells, inflammation and proliferation of smooth muscle cells. SiOtud6b can knock down the expression of Otud6b protein in endothelial cells and smooth muscle cells, and reverse the effect of rOtud6b.

In this study, we also noted an increase of Otud6b in the cytoplasm of HPAECs during hypoxia. In the cytoplasm, Otud6b interacts with HIF, which mediates the cell’s transcriptional response to hypoxia. HIF-1α drives the initial response to hypoxia, while HIF-2α drives the chronic hypoxia response [36]. Thus, Otud6b can modulate acute and chronic hypoxia responses. Although this study did not specifically examine whether the activation of HIF itself through hypoxia is regulated by Otud6b, we found that activation of Otud6b expression with rOtud6b does increase HIF expression, which further regulates the transcription of cytokines and important proteins (such as VEGF, ET-1, etc.), which contribute to the development of PAH.

We further discussed the regulatory relationship between Otud6b and HIF-1α. Liu et al. [37] reported that the stability of HIF-1α in hepatocellular carcinoma (HCC) was controlled by Hippel–Lindau (pVHL)-mediated ubiquitination. Otud6b can directly bind pVHL, reduce pVHL ubiquitination and proteasome degradation, and reduce HIF-1α accumulation in HCC cells under hypoxia conditions. However, Otud6b limits ubiquitination of pVHL independently of its deubiquitase activity. Otud6b couples pVHL and elongin B/C to form more CBC^VHL^ ligase complex that protect pVHL from proteasome degradation. Therefore, we conclude that Otud6b regulates HIF-1α in a non-proteasome-dependent manner. At the same time, Liu et al. [35] also found that Otud6b gene is a direct transcription target of HIF-1α, which is up-regulated under hypoxia conditions. Therefore, we conclude that Otud6b is not regulated by HIF-1α transcription.

Calpain-1 is important in PAH development [38, 39]. Previous studies in our laboratory have shown that Calpain-1 mediates vascular remodeling and fibrosis with hypoxia pulmonary hypertension through HIF-1α [20]. In this study, we found that Calpain-1 and Outd6b are mutually regulated. Calpain-1 KO mice and Calpain-1 inhibitor MDL inhibited Calpain-1 protein expression, decreased Otud6b protein expression, and further improved PAH. Activation of Otud6b protein expression with rOtud6b reversed the regulation of Calpain-1 KO and MDL and exacerbated the progression of PAH.

Interestingly, our experimental results show that HIF-1α and Calpain-1/Otud6b regulate each other through positive feedback. Calpain-1 modulates the expression of HIF-1α in HPAECs exposed in hypoxia. Similarly, HIF-1α regulates the expression of Calpain-1 during hypoxia. At the same time, we also found that rOtud6b induced to increased expression of Calpain-1 and HIF-1α. In HPAECs, Otud6b itself increases the expression of Calpain-1 during hypoxia, which may be related to the activation of HIF-1α by hypoxia. However, more research is needed to determine the mechanisms of this interaction. But, our experimental results suggest that Otud6b can “trigger” the overactivation of HIF-1α in hypoxia-induced HPAECs, increasing the transcriptional activity of HIF-1α. Therefore, we believe that there is a positive feedback loop between HIF-1α and Calpain-1/Otud6b expression, which further enhances or amplifies pathological signals and processes.

Of course, there are limitations to the study. For example, we only modelled PAH at animal and cellular levels and did not obtain tissue samples from PAH patients. However, we also demonstrate for the first time that Otud6b is an important regulator in the treatment of PAH, and that regulating the expression of Otud6b protein has both a palliative and therapeutic effect on PAH. Therefore, these findings may provide a potential therapeutic strategy for Otud6b as a therapeutic target for PAH.

## Materials and methods

### Animal experiments

All animal operations were carried out in accordance with the principles approved by the Animal Ethics Committee of Jinzhou Medical University. The Calpain-1 gene knockout mice is derived from the C57BL/6 N strain and is a complete knockout of thecalpain-1 gene, generated by Cyagen Biosciences. C57BL/6 mice and Calpain-1 gene knockout mice weighing 18–22 g were randomly divided into the following nine groups (*n* = 12 for each group): (a) the Normoxia group (Nor), (b) the KO Normoxia group (KO Nor), (c) the Hypoxia group (Hyp), (d) the KO Hyp group (KO Hyp), (e) the KO Hyp group + rOtud6b (KO Hyp + rOtud6b), (f) the Nor + siNC group (Nor + siNC), (g) the Nor + siOtud6b group (Nor + siOtud6b), (h) the Hyp + siNC group (Hyp + siNC), (i) the Hyp + siOtud6b group (Hyp + siOtud6b). Mice in the normoxia group were exposed to a normal environment containing 21% O_2_, while mice in the hypoxia group were exposed to an atmospheric chamber containing 10% O_2_ for 4 weeks. During the feeding period, each group of mice drank and eat freely.

### Hemodynamic and weighing methods

After 4 weeks, the mice were anesthetized by intraperitoneal injection of pentobarbital sodium (20%, US, Sigma). A pressure sensor was inserted into the right external jugular vein and into the right ventricle and pulmonary arteries to measure right ventricular systolic pressure (RVSP) and mean pulmonary artery pressure (mPAP). The weight method measures the ratio of right ventricular weight to left ventricular weight, RV/(LV + s). The mice were euthanized, left lung tissues was collected, stored at -80 ℃ until use, and the remaining lung tissues was fixed with formalin.

### H&E staining

The left lung tissues of mice was isolated, fixed in 4% paraformaldehyde for 24 h, and embedded in paraffin. Cut the tissue into 5 µM pieces sheet. Hematoxylin eosin (H&E) staining was used to detect the vascular wall area ratio (WA%) and vascular wall thickness ratio (WT%).

### Immunohistochemical staining

After the sections were infiltrated with xylene and dewaxed with alcohol, antigen repair was performed to eliminate peroxidase activity and block nonspecific binding. The slide was incubated overnight with anti Otud6b, Ki-67 and Caspase-3 (1:100) primary antibodies at 4 °C, and then bound to the secondary antibody. The slides were stained with DAB and hematoxylin and observed.

### Echocardiography

Transthoracic echocardiography was performed through the UBM system (Esaote, Sigma PVET). Mice were anesthetized and maintained under 1–3% isoflurane during the procedure. Echocardio graphic measurements were performed by a blinded investigator and were conducted at the mid-papillary muscle level, as guided by twodimensional long-axis images. Pulmonary artery acceleration time (PAT), ejection time (ET), and the ratio of PAT/ET were measured and calculated with Esaote Analysis software. PAT is the time interval from the beginning of the pulmonary artery inflow to the peak velocity recorded by pulse Doppler, and ET is the time interval from the beginning to the end of the pulse systolic blood flow. PAT, ET and PAT/ET were used to evaluate pulmonary artery pressure in rats indirectly.

### RT-qPCR

Total RNA was extracted from HPAECs, HPSAMC and the mice aorta with Trizol reagent. Complementary DNA (cDNA) was reverse transcribed from total RNA samples using ABScript II RT Master Mix (ABclonal). RT-qPCR was performed using Genious 2X SYBR Green Fast qPCR Mix (ABclonal) with ACTB being the loading control. GenScript Biotech™ synthesized all primers. There are the primer sequences in Table S1.

### Proteomic analysis

During the 4 weeks of hypoxia modeling, lung tissues samples from Nor, KO Nor, Hypoxia and KO Hyp mice were collected, and total proteins were extracted from the lung tissues. They were subjected to trypsin hydrolysis, TMT labeling, and HPLC classification. Finally, they were analyzed by liquid chromatography mass spectrometry.

### Immunofluorescence

The slide/24 wells cell plate was infiltrated into PBS containing 0.5% Triton X-100 for 30 min and incubated in PBS containing 5% bovine serum albumin for 30 min. The slide and anti-alpha smooth muscle Actin (α-SMA), Otud6b, Ki-67, and Caspase-3 (1:100) were incubated overnight at 4 ℃. On the second day, a slide/24 well cell plate was incubated with a HRP second antibody bound to fluorescein isothiocyanate (FITC) and a DAPI staining solution, and then observed under a fluorescence microscope.

### HPAECs and HPASMCs culture

Human pulmonary artery endothelial cells (HPAECs) and human pulmonary artery smooth muscle cells (HAPSMCs) were purchased from BLUEFBIO (Shanghai, China) and cultured in containing 10% FBS and 100 U/ml penicillin/streptomycin endothelial cell growth supplement (ECGS) at 37 ℃ and 5% CO_2_. After that, they were incubated in a 3% oxygen concentration atmospheric pressure hypoxia chamber for 24 h to induce an in vitro model.

### Transfection of siRNA

The siRNA targeting Otud6b (GGGAATGAAGAACGCCGTT) and the non-targeted negative control siRNA (siNC) were designed and synthesized by Heyuan Biotechnology (Shanghai). Transfection with the above siRNA (80ul) according to the manufacturer’s instructions. After 21 days, the transfection of animals was observed. Transfection efficiency was verified by Western blots (> 85%). Each siRNA assay was validated with three different specific siRNA and three different NC siRNA.

### Analysis of apoptosis by flow cytometry

HPAECs were collected 48 h after transfection with siRNA control or siOtud6b under normal or hypoxia conditions. The cells are trypsinized and suspended with a buffer. About 5µL Annexin V-FITC was further incubated at room temperature. Specimens were analyzed by flow cytometry ten minutes later.

### Western blot

The collected lung tissues, HPAECs and HPASMCs were homogenized in RIPA lysis buffer. The protein concentration was measured using the BCA protein analysis kit. The samples were separated by SDS-PAGE (10% polyacrylamide gel) and transferred to PVDF membrane. Seal the membrane with 1% BSA for 1 h, and at 4 ℃, compare it with anti Tumor Necrosis Factor-alpha (TNF-α, 1:1000, ABclonal, A24214), anti Interleukin-1 beta (IL-1β, 1:1000, ABclonal, A16288), anti Interleukin-6 (IL-6, 1:1000, ABclonal, A0286), anti OTU Domain Containing 6B (Otud6b, 1:5000, Proteintech, 25430-1-AP), anti Calpain-1 (1:10000, Proteintech, 10538-1-AP), anti Endothelin-1 (ET-1, 1:1000, ABclonal, A0686), anti Vascular Endothelial Growth Factor (VEGF, 1:1000, ABclonal, A23759), anti Hypoxia inducible factor 1 (HIF-1α, 1:1000, ABclonal, A11945) and anti β-action (1:100000, ABclonal, AC026) were mixed overnight. The membrane was washed three times with TBST, and then incubated with a second antibody binding HRP (1:10000) at room temperature for 1 h. Develop using ECL developer.

### Data analysis

The data were expressed as an average ± SEM and analyzed using SPSS 25.0. One-way analysis of variance was used for comparison between groups, multiple comparisons and homogeneous subset test were conducted afterwards. *P* < 0.05 is considered statistically significant.

### Electronic supplementary material

Below is the link to the electronic supplementary material.

Supplementary Material 1

Supplementary Material 2

Supplementary Material 3

## Data Availability

The datasets generated during and/or analysed during the current study are not publicly available due to this article has not yet been published but are available from the corresponding author on reasonable request.
