# Supplementary material for: Otud6b induces pulmonary arterial hypertension by mediating the Calpain-1/HIF-1α signaling pathway
Source: Cell Mol Life Sci. 2024 Jun 15;81(1):258. doi: 10.1007/s00018-024-05291-3 (PMC11335297; doi:10.1007/s00018-024-05291-3)
Supplement: Supplementary file 3 — Supplementary Material 3 [file 18_2024_5291_MOESM3_ESM.docx]

**Table S1**

Primer sequences.

Gene Primer sequences (5’-3’)

hOTUD6B Forward: TGAGAAGGCATCGCAAAGAGA

Reverse: ATCTTCGGTGAGTTGCTTCCT

mOtud6b Forward: AGCTCACGGAAGATGTTGCTA

Reverse: TTTTGTGCTTTTGAAATCCGAGG
